# Supplementary material for: Covariance patterns between sleep health domains and distributed intrinsic functional connectivity
Source: Nat Commun. 2023 Nov 6;14:7133. doi: 10.1038/s41467-023-42945-5 (PMC10628193; doi:10.1038/s41467-023-42945-5)
Supplement: Supplementary file 1 — Supplementary Information [file 41467_2023_42945_MOESM1_ESM.pdf]

Covariance patterns between sleep health domains and distributed intrinsic functional connectivity

Supplemental materials

Supplemental Methods

Participants in the external classification dataset

The external classification dataset(dataset5) initially involved 39 ID patients and 35 HCs. All the participants in the classification dataset5 received the MRI scanning at the Second Hospital of Hebei Medical University. Patients with insomnia disorder were diagnosed by experienced hospital psychiatrists (J.Y. L. and N.H.H) according to the DSM-V and insomnia symptoms has lasted at least three nights a week for more than 3 months. The exclusion criteria were as follows: (1) other comorbid mental disorders, (2) serious neurological or medical diseases, (3) other sleep disorders, (4) frequent jet lag, (5) Epworth Sleepiness Scale (ESS) score > 11, (6) Beck Depression Index (BDI) score > 20 or Beck Anxiety Index (BAI) score > 45, (7) pregnancy, lactation, or plans to become pregnant during the study period, (8) structural brain abnormalities based on magnetic resonance imaging (MRI), and (9) use of psychiatric medication, hypnotics, or having been treated with CBT-I in the last 2 weeks. All the health controls met the following criteria: (1) Aged 18–65 years, (2) righthanded, (3) no symptoms or history of psychiatric disorders and sleep disorders, (4) a total score <5 on the PSQI at screening and a total score <8 on the ISI at screening, and (5) not taking any psychotropic medications or hypnotics during their lifetime. Participants with any findings of pathological brain MRI as well as ineligibility for MRI scanning (any type of metal implant) were excluded in the study. Four participants were excluded due to excessive head motion during the scanning (e.g., with a mean FD larger than 0.3 mm), resulting in a final sample of 35 ID patients (mean age= 45.25 years old, SD= 11.28) and 35 HC (mean age=40.83 years old, SD= 9.27). Participants were compensated for 80 Chinese Yuan for their participation. The research projects were approved by the review boards of the Second Hospital of Hebei Medical University, and written informed consent was obtained from each participant in accordance with the Declaration of Helsinki. The two groups were matched for age, sex, and head motion (Table S6). The participants' demographic characteristics are summarized in Table S6.

Data acquisition and image preprocessing

The External Classification sample:

Data acquisition

All MR Images in the external classification dataset were acquired by using a Philips Achieva 3.0T scanner (Philips Healthcare, Netherlands) with an 8-channel phased-array head coil located at the Second Hospital of Hebei Medical University. In order to minimize motion, prior to data acquisition participants' heads were stabilized in the head coil using one foam pad over each ear and a third over the top of the head. High- resolution T1- weighted anatomical images were collected using a 3D Fast Field Echo (FFE) sequence (TR = 6.3 ms, TE = 2.8 ms, FOV = 256 × 256 mm^2, thickness = 1 mm, voxel size = 1 × 1 × 1 mm^3, resolution matrix = 256 × 256, slices = 176). Approximately eight minutes of rs-fMRI data containing 245 volumes were acquired for each subject using a sagittal T2-weighted Single echo (SE) sequence (TR/TE = 2000/80 ms, flip angle = 90°, voxel size = 3.5 ×3.5 ×3.5 mm^3, FOV = 192 × 192 mm^2, axial slices = 41). The first four volumes were discarded to ensure steady- state longitudinal magnetization. During the resting-state scanning, participants were instructed to fix on a crosshair in the center of black background screen without thinking intentionally in the mind and keep as motionless as possible.

Image preprocessing

The preprocessing steps on all collected neuroimaging data in the external classification dataset were performed using fMRIPrep 21.0.1[12] (RRID:SCR_016216), which is based on Nipype 1.6.1[3](RRID:SCR_002502). Details of the structural and functional data preprocessing can be found in the follow-up supplementary materials.

Image preprocessing using fMRIPrep for the classification dataset

Anatomical data preprocessing

A total of 1 T1-weighted (T1w) images were found within the input BIDS dataset. The T1-weighted (T1w) image was corrected for intensity non-uniformity (INU) with N4BiasFieldCorrection[4], distributed with ANTs 2.3.3 [5] (RRID:SCR_004757), and used as T1w-reference throughout the workflow. The T1w-reference was then skull-stripped with a Nipype implementation of the antsBrainExtraction. sh workflow (from ANTs), using OASIS30ANTs as target template. Brain tissue segmentation of cerebrospinal fluid (CSF), white-matter (WM) and gray-matter (GM) was performed on the brain-extracted T1w using fast (FSL 6.0.5.1:57b01774, RRID:SCR_002823 [6]). Volume-based spatial normalization to two standard spaces (MNI152NLin6Asym, MNI152NLin2009cAsym) was performed through nonlinear registration with antsRegistration (ANTs 2.3.3), using brain-extracted versions of both T1w reference and the T1w template. The following templates were selected for spatial normalization: FSL's MNI ICBM 152 non-linear 6th Generation Asymmetric Average Brain Stereotaxic Registration Model [[7] RRID:SCR_002823; TemplateFlow ID: MNI152NLin6Asym], ICBM 152 Nonlinear Asymmetrical template version 2009c [[8], RRID:SCR_008796; TemplateFlow ID: MNI152NLin2009cAsym].

Functional data preprocessing

For each of the 1 BOLD runs found per subject (across all tasks and sessions), the following preprocessing was performed. First, a reference volume and its skull-stripped version were generated using a custom methodology of fMRIPrep. Head-motion parameters with respect to the BOLD reference (transformation matrices, and six corresponding rotation and translation parameters) are estimated before any spatiotemporal filtering using mcflirt (FSL 6.0.5.1:57b01774,[9]). BOLD runs were slice-time corrected to 0.958s (0.5 of slice acquisition range 0s-1.92s) using 3dTshift from AFNI ([10], RRID:SCR_005927). The BOLD time-series (including slice-timing correction when applied) were resampled onto their original, native space by applying the transforms to correct for head-motion. These resampled BOLD time-series will be referred to as preprocessed BOLD in original space, or just preprocessed BOLD. The BOLD reference was then co-registered to the T1w reference using mri_coreg (FreeSurfer) followed by flirt (FSL 6.0.5.1:57b01774, [11]) with the boundary-based registration [12] cost-function. Co-registration was configured with six degrees of freedom. Several confounding time-series were calculated based on the preprocessed BOLD: framewise displacement (FD), DVARS and three region-wise global signals. FD was computed using two formulations following Power (absolute sum of relative motions, [13]) and Jenkinson (relative root mean square displacement between affines, [9]). FD and DVARS are calculated for each functional run, both using their implementations in Nipype (following the definitions by [13]). The three global signals are extracted within the CSF, the WM, and the whole-brain masks. Additionally, a set of physiological regressors were extracted to allow for component-based noise correction (CompCor, [14]). Principal components are estimated after high-pass filtering the preprocessed BOLD time-series (using a discrete cosine filter with 128s cut-off) for the two CompCor variants: temporal (tCompCor) and anatomical (aCompCor). tCompCor components are then calculated from the top 2% variable voxels within the brain mask. For aCompCor, three probabilistic masks (CSF, WM and combined CSF+WM) are generated in anatomical space. The implementation differs from that of Behzadi et al. in that instead of eroding the masks by 2 pixels on BOLD space, the aCompCor masks are subtracted a mask of pixels that likely contain a volume fraction of GM. This mask is obtained by thresholding the corresponding partial volume map at 0.05, and it ensures components are not extracted from voxels containing a minimal fraction of GM. Finally, these masks are resampled into BOLD space and binarized by thresholding

at 0.99 (as in the original implementation). Components are also calculated separately within the WM and CSF masks. For each CompCor decomposition, the k components with the largest singular values are retained, such that the retained components' time series are sufficient to explain 50 percent of variance across the nuisance mask (CSF, WM, combined, or temporal). The remaining components are dropped from consideration. The head-motion estimates calculated in the correction step were also placed within the corresponding confounds file. The confound time series derived from head motion estimates and global signals were expanded with the inclusion of temporal derivatives and quadratic terms for each [15]. Frames that exceeded a threshold of 0.5 mm FD or 1.5 standardised DVARS were annotated as motion outliers. The BOLD time-series were resampled into standard space, generating a preprocessed BOLD run in MNI152NLin6Asym space. First, a reference volume and its skull-stripped version were generated using a custom methodology of fMRIPrep. All resamplings can be performed with a single interpolation step by composing all the pertinent transformations (i.e. head-motion transform matrices, susceptibility distortion correction when available, and co-registrations to anatomical and output spaces). Gridded (volumetric) resamplings were performed using antsApplyTransforms (ANTs), configured with Lanczos interpolation to minimize the smoothing effects of other kernels [16]. Non-gridded (surface) resamplings were performed using mri_vol2surf (FreeSurfer).

Many internal operations of fMRIPrep use Nilearn 0.8.1 ([17], RRID:SCR_001362), mostly within the functional processing workflow. For more details of the pipeline, see the section corresponding to workflows in fMRIPrep's documentation.

Consistent with BBP dataset, the processed functional images were further smoothed, underwent regression of motion and non-relevant signals, including linear trend, Friston 24 head motion parameters, white matter (CompCor, 5 principal components), and CSF signal (CompCor, 5 principal components), scrubbed and filtered.

Copyright Waiver
The above boilerplate text was automatically generated by fMRIPrep with the express intention that users should copy and paste this text into their manuscripts unchanged. It is released under the CC0 license.

Partial least squares analysis

PLS is a multivariate procedure that seeks maximal correlations between two matrices by deriving LVs, which are optimal linear combinations of the original matrices [18,19]. We applied PLS to the rsFC and behavioral measures spanning multiple domains of sleep health in the discovery dataset from BBP sample. Given two matrices, X_{n*p} and $Y_{n\times q}$, where n is the number of observations (e.g., participants, here n=687), p and q are the number of variables (e.g., RSFC (p=30,135) and behavioral features (q=36), respectively), after z-scoring X and Y (across participants), we calculated the covariance matrix $R=Y^TX$. Then, singular value decomposition (SVD) of $R=USV^T$ produced in three low-dimensional latent variables: U and V are the singular vectors (called SH and FC saliences, similar to loadings in principal components analysis), while S is a diagonal matrix containing the singular values. After that, by linearly projecting the FC and SH measures of each participant onto their respective saliences, we obtained individual-specific FC and SH composite scores for each LV, which reflect the participants' individual FC and behavioral contribution to each LV (similar to factor scores in principal components analysis). PLS seeks to find saliences that maximize the covariance between FC and behavioral composite scores. The covariance explained by each LV is estimated by dividing the squared singular value by the sum of all squared singular values. Before the PLS analysis, we regressed out the confounding effects from both FC and behavior data including mean FD, age, and sex.

More details about the inference and validation of the statistical model using nonparametric methods were provided below:

In order to evaluate the statistical significance of the LVs, we applied permutation testing using 5000 permutations for behavioral data and repeating the PLS analysis to determine the null distribution of the singular values.

To assess the contribution of a given connection (FC) or behavior to a given LV, we computed correlations between the original measure (connection or behavior) and the corresponding composite scores[20][21]. A large correlation value (i.e., large weight, positive or negative) for a given measure (behavioral or connection) for a given LV indicates greater contribution of the behavior or connection to the LV. Then, the confidence intervals for these correlations were determined a by bootstrapping procedure that generated 5000 samples with replacement from the original FC and SH data.

To identify variables (connection or SH measures) that make a significant contribution to the overall pattern, we calculated Bootstrapped Z scores as the ratio of each variable's correlation coefficient (i. e., weight) to its bootstrap-estimated standard error. Then, we converted the Z scores to p values, which were FDR corrected ($q < 0.05$). In the PLS literature, the bootstrap method is utilized to identify elements that exhibit consistent experimental effects. This approach eliminates the need for adjustments in case of multiple comparisons, as no statistical test is conducted [22]. However, our objective was not only to ascertain the reliability and stability of the z-scores but also to determine their statistical significance. Therefore, we performed individual tests for each z-score, resulting in the requirement for corrections to account for multiple comparisons.

To test the generalizability of each LV, we used a 10-fold cross-validation of the PLS analysis with 200 repetitions. Importantly, the cross-validation approach can help to guard against overfitting that arises from high dimensional neurobiological data [23]. Specifically, first, we assigned 90 % of the participants to the training set and the remaining 10 % of participants to the test set. For each training set, PLS was used to estimate connection and behavioral saliences (i.e., Utrain and Vtrain). Next, the test data were projected onto the connection and behavioral patterns derived from the training set. This allowed us to estimate individual-specific connection and behavior composite scores and their correlation for the test sample (i.e. corr(XtestUtrain, YtestVtrain)) for LVs. This procedure was repeated 200 times to make sure the results are not biased by the initial split. It should be noted that, to avoid data leakage issue, the adjustment for confounds and data standardization and were performed within the cross-validation loop (i.e., at first, we estimated parameters of data standardization and confounds regression in the nine training folds and then applied the obtained parameters to the test fold). Finally, we used a permutation test (behavioral data shuffled 1000 times) to assess the significance of these correlation coefficients.

False discovery rate (FDR) correction ($q < 0.05$) was applied to all analyses.

Notably, there are two popular approaches, i.e., canonical correlation analysis (CCA) and PLS, to implement multivariate covariance. We chose PLS over CCA because we noted that CCA applies SVD to $(Y'Y)^{-1/2} Y'X (X'X)^{-1/2}$, so when the number of features is more than the number of samples, $Y'Y$ and/or $X'X$ become rank deficient, so matrix inversion becomes problematic[24]. However, there was no consensus on how to solve this issue with CCA. Since PLS applies SVD to $Y'X$, this is not an issue for PLS. Moreover, considering that our cross-validation results were already successful (mean $r = 0.17$, range of permuted p [$2.0\text{x}10^{-3}$, $3.0\text{x}10^{-3}$]), we opted not to perform any dimensionality reduction or to add any regularization constraint to avoid

additional tuning of hyperparameters (e.g., number of principal components, level of sparsity, etc.) that generally requires larger sample size and have higher computational costs.

Supplemental Results

The robustness of the obtained LV1 was further ensured using a different Seitzman et al' Atlas containing 300 regions for the RSFC construction (see **Methods** for the details). To do this, we replicated the PLS procedure conducted in the discovery dataset. The LV1 accounted for 23.9% variance and still survived after permutation testing with FDR correction (q<0.05) in the replication dataset with significant association (r =0.52, permuted p = 0.02) between RSFC and behavioral composite scores (figure S1). Importantly, the obtained LV1 was largely replicated, evidenced by the high correlation (r = 0.99 for behavior data; r = 0.78 for RSFC data) between loading scores of Brainconnectome Atlas and Seitzman et al' Atlas, and the moderate to high correlation (r =0.99 for behavior data; r = 0.59 for RSFC data) between salience scores of Brainconnectome Atlas and Seitzman et al' Atlas (figure S2). It should be noted that we calculated r value for RSFC loading and salience only within the overlapped 167 regions, i.e., 13,861 edges in Brainconnectome Atlas and Seitzman et al' Atlas.

Table S1. Sleep questions and sleep health domains

Domains of sleep health	Measures	Scale/ Questionnaire /Item	Explanations of the responses	Definition of "poor" sleep health
**Satisfaction with sleep/ Sleep quality**	Not get enough sleep	In the last month, have you slept enough?	5-point-rating, the higher the score, the less the subjects felt they were getting enough sleep	Sleep is far from enough
	Feelings from wake-up	In the last month, have you woken up feeling well rested？	5-point-rating, the higher the score, the less the subjects felt well rested from waking up	Not feel well rested
	Necessity of nap	Do you feel the need for a nap?	5-point-rating, the higher the score, the more necessary the subjects felt to take a nap	Nap is highly necessary
	Needed nap time	How long do you think a nap is appropriate if you have enough time and appropriate environment for sleep?	5-point-rating, the higher the score, the subjects needed more nap time	Long nap time is needed
	PSQI-T (otal score)	The Pittsburgh Sleep Quality Index (PSQI; Buysse et al., 1989)<br>Total score	PSQI is a 19-item questionnaire that measures self-reported sleep quality over the last month, with higher scores indicating poorer sleep quality	PSQI > 7
	Subjective sleep quality	A component from PSQI measuring subjective sleep quality	4-point scale (responses range from 0 to 3), the higher the score, the poorer subjective sleep quality	Poor subjective sleep quality
**Alertness during waking hours**	Epworth sleepiness scale	Epworth sleepiness scale (ESS; Johns, 1991) is an 8-item questionnaire that measures the general level of daytime sleepiness.	4-point scale (response range from 0 to 3), the higher the score, the higher likelihood to fall asleep during eight different situations	ESS >11
	Mind-wandering	Mind-Wandering Questionnaire (MWQ; Mrazek, Phillips, Franklin, Broadway, & Schooler, 2013) is a self-report measure consisting of five items assessing overall mind wandering tendencies.	Items are answered on a six-point rating scale ranging from 1 = Almost never to 6 = Almost always. Mean scores are reported where a higher score indicates more tendency to mind wander.	High tendency to mind wandering
	Spontaneous mind wandering	"Mind-wandering: Spontaneous" (MW-S; Carriere, Seli, & Smilek, 2013) scale is a 4-item questionnaire used to index spontaneous mind-wandering. Sample item for MWS is: "I find my thoughts wandering spontaneously.	7-point scale, the higher the score, the more spontaneous mind-wandering. Mean scores are reported.	High spontaneous mind-wandering
	Deliberate mind wandering	"Mind-wandering: Deliberate" (MWD; Carriere, Seli, & Smilek, 2013) scale is a 4-item questionnaire used to index deliberate mind-wandering. Sample item for MWD is: "I allow my thoughts to wander on purpose".	7-point scale, the higher the score, the more deliberate mind-wandering. Mean scores are reported.	Undetermined

	Attention-related cognitive errors	Attention-Related Cognitive Errors Scales (ARCES; Cheyne et al. 2006) is a 12-item questionnaire used to assess the frequency of attention-related cognitive errors in individuals	5-point scale, the higher the score, the higher the frequency of attention-related cognitive errors	High frequency of attention-related cognitive errors
	Frequency of daydream	The Daydreaming Frequency Scale (DDFS; Giambra, 1993) is a 12-item questionnaire addressing the frequency of daydreaming in everyday life. A sample item is: "I lose myself in active daydreaming"	Each item is answered on a 5-point scale (0–4) indicating frequency. The sum score can range from 0 to 48 where higher scores indicate higher frequency of daydreaming in everyday life.	High occurrence of task-unrelated thoughts
	Fatigue severity	Fatigue severity scale (FSS; Krupp et al. 1989) is a 9-item questionnaire used to assess the effect of fatigue on daily functioning	7-point scale, the higher the score, the bigger impact of fatigue on daily functioning	High fatigue severity
	Physical fatigue	"Chalder Fatigue Scale (CFS;Chalder et al. 1993): physical fatigue" is an 8-item scale to assess the severity of physical fatigue	Yes/No response, the higher the score, the more severity of physical fatigue	High physical fatigue severity
	Mental fatigue	"Chalder Fatigue Scale (CFS; Chalder et al. 1993): mental fatigue" is a 6-item scale to assess the severity of physical fatigue	Yes/No response, the higher the score, the more severity of mental fatigue	High mental fatigue severity
	Valid sleep cue RT	The reaction time (RT) for the valid sleep cues in the modified Posner paradigm (Woods et al 2009)*.	When the target is presented in the box on the same side as the cue, this is considered a valid trail. The RT was recorded	Undetermined
	Invalid sleep cue RT	The reaction time (RT) for the invalid sleep cues in the modified Posner paradigm (Woods et al 2009).	When the target is presented in the box on the other side as the cue, this is considered an invalid trail. The RT was recorded	Undetermined
	Valid sleep cue ACC	The accuracy (ACC) for the valid sleep cues in the modified Posner paradigm (Woods et al 2009).	When the target is presented in the box on the same side as the cue, this is considered a valid trail. The ACC was recorded	Undetermined
	Invalid sleep cue ACC	The accuracy (ACC) for the invalid sleep cues in the modified Posner paradigm (Woods et al 2009).	When the target is presented in the box on the other side as the cue, this is considered an invalid trail. The ACC was recorded	Undetermined
**Timing of sleep**	Morningness-eveningness questionnaire	The Morningness-Eveningness questionnaire (MEQ; Horne & ¨Ostberg, 1976) is a widely employed 19-item self-report questionnaire addressing daily preferences for practicing physical and mental activities and the subjective alertness after wake time and before bedtime	Items are answered on a four- or five-point scale. The sum gives a score ranging from 16 (clear eveningness) and 86 (clear morningness). Evening type: from 16 to 52; Intermediate type: from 53 to 64; and Morning type: from 65 to 86	Evening type
**Sleep efficiency/ continuity**	Sleep efficiency	Sleep efficiency refers to the	the total sleep time (TST) divided by the time in bed (TIB)	Low sleep efficiency

		ratio between the time spent asleep and the time available for sleeping.		
	Sleep latency	A component from PSQI measuring the time it takes for an individual to go from being fully awake to sleeping. The question for this item is: “During the past month, how long (in minutes) has it usually takes you to fall asleep each night?”	If it takes less than (<)15 minutes to fall asleep, the score is 0; If it takes 16-30 minutes, the score is 1; If it takes 31-60 minutes, the score is 2; if it takes more than (>)60 minutes, the score is 3.	The higher the score, the poorer the sleep health
	Wake-up times	An item from the sleep diary to ask an individual; “how many times did you wake up last night?”	The higher the score, the higher the number of night awakenings	High wake-up times
**Sleep duration**	Total sleep time	During the past month, how many hours of actual sleep did you get at night?	Consensus statements from several scientific, medical, and public health organizations recommend that adults, in general, should obtain at least 7 hours of sleep per night	undetermined
**Sleep deficiency**	Insomnia Severity Index	The Insomnia Severity Index (ISI; Bastien et al., 2001)) is a brief 7-item screening measure of insomnia.	Items are rated on a 5-point Likert scale ranging from 0 to 4. The higher ratings always correspond to higher sleep related difficulties	High sleep related difficulties
	Hyperarousal	The hyperarousal scale (HAS; Pavlova et al. 2001) consists of 26 items that assess the hyperarousal behavioral trait.	Items are rated on a 4-point Likert scale ranging from 0 to 3. The higher ratings correspond to higher arousing levels.	High hyperarousal levels
	Ford insomnia response to stress test	Ford Insomnia Response to Stress Test (FIRST; Drake et al. 2004) is a self-report tool that measures sleep reactivity (i.e., vulnerability to experience situational insomnia under stressful conditions), which consists of 9 items.	Items are rated on a 4-point Likert scale ranging from 1 to 4. Higher scores indicate greater insomnia responses to stressful events	Total score > 16
	Sleep disturbances	A component from PSQI measuring sleep disturbances which consists of 9 questions.	If the sum of the nine question scores is 0, the component score is 0; if the sum is 1-9, the component score is 1; if the sum is 10-18, the sum is 2; if the component is 19-27, the sum is 3	The higher the score, the poorer the sleep health
	Use of sleep medications	A component from PSQI measuring use of sleep medications. The question is:” During the past month, how often have you taken medicine to help you sleep (prescribed or “over the counter”)?”	If the answer is “Not during past month”, the score is 0; if the answer is “Less than once a week”, the score is 1; if the answer is “Once or twice a week”, the score is 2; if the answer is “Three or more times a week”, the answer is 3	The higher the score, the poorer the sleep health
	Daytime dysfunction	A component from PSQI measuring daytime dysfunction which consists of two questions: “7. During the past month, how often have you had	If the Sum of Q7 and Q8 sub scores is 0, the component score is 0; if the sum is 1-2, the component score is 1;	The higher the score, the

		trouble staying awake while driving, eating meals, or engaging in social activity?"; "8. During the past month, how much of a problem has it been for you to keep up enough enthusiasm to get things done?"	if the sum is 3-4, the component score is 2; if the sum is 5-6, the sum is 3	poorer the sleep health
**Sleep beliefs, attitudes, and habits**	DBAS-total score	The original Dysfunctional beliefs and attitudes about sleep scale (DBAS; Morin 1993) is a 30-item self-report questionnaire designed to identify and assess various sleep/insomnia-related cognitions (e.g., beliefs, attitudes, expectations, appraisals, attributions). The DABS consists of five dimensions.	Items are rated on a 11-point Likert scale ranging from 0 to 10. Higher total scores indicate more dysfunctional beliefs and attitudes about sleep	undetermined
	Misconceptions about causes	A dimension of the DABS measuring the misconceptions about causes of insomnia (e.g., "I believe insomnia is essentially the result of a chemical imbalance").	Items are rated on a 11-point Likert scale ranging from 0 to 10. Higher dimension score indicates more misconceptions about causes of insomnia	undetermined
	Diminished control and predictability	A dimension of the DABS measuring the diminished perception of control and predictability of sleep (e.g., "When I sleep poorly on one night, I know it will disturb my sleep schedule for the whole week")	Items are rated on a 11-point Likert scale ranging from 0 to 10. Higher dimension score indicates more diminished perception of control and predictability of sleep	undetermined
	Unrealistic sleep expectations	A dimension of the DABS measuring the unrealistic sleep expectations (e.g., "I must get 8 hours of sleep to feel refreshed and function well the next day")	Items are rated on a 11-point Likert scale ranging from 0 to 10. Higher dimension score indicates more unrealistic sleep expectations	undetermined
	Misattribution of the consequences	A dimension of the DABS measuring the misattribution or amplification of its consequences (e.g., "I am concerned that chronic insomnia may have serious consequences on my physical health").	Items are rated on a 11-point Likert scale ranging from 0 to 10. Higher dimension score indicates more misattribution or amplification of its consequences.	undetermined
	Faulty beliefs about sleep promoting practices	A dimension of the DABS measuring faulty beliefs about sleep-promoting practices (e.g., "When I have trouble sleeping, I should stay in bed and try harder").	Items are rated on a 11-point Likert scale ranging from 0 to 10. Higher dimension score indicates more faulty beliefs about sleep-promoting practices.	undetermined

Note. *The modified Posner paradigm implemented in the BBP in accordance with the experiment conducted by Woods and associates[25].

Table S2. Absolute correlations between RSFC (or behavioral) saliences obtained in control analyses and sleep health saliences from the original PLS analysis.

	GSR	Total intracranial volume regression	Hour of acquisition regression	pre-scanning positive and negative affect regression	BMI regression	Family income regression	Confounds included	Behavior normalized
Correlations with original RSFC saliences	0.97	0.99	0.99	0.99	0.99	0.99	0.96	0.98
Correlations with original behavioral saliences	0.93	0.99	0.99	0.96	0.99	0.99	0.92	0.99

Note. GSR, global signal regression; BMI, body mass index; RSFC, resting state functional connectivity.

Table S3. Top five nodes with highest degree in the positive and negative network.

Regions	Anatomical and modified Cyto-architectonic descriptions	Networks	Label	MNI (x, y, z)
**Positive Network**				
INS, Insular Gyrus / INS_L_6_6	dId, dorsal dysgranular insula	VAN	173	-38, 5, 5
INS, Insular Gyrus / INS_R_6_6	dId, dorsal dysgranular insula	VAN	174	38, 5, 5
PrG, Precentral Gyrus / PrG_L_6_5	A4tl, area 4(tongue and larynx region)	VAN	61	-52, 0, 8
PrG, Precentral Gyrus / PrG_R_6_5	A4tl, area 4(tongue and larynx region)	VAN	62	54, 4, 9
PrG, Precentral Gyrus / PrG_R_6_6	A6cvl, caudal ventrolateral area 6	DAN	64	51, 7, 30
**Negative Network**				

Tha, Thalamus / Tha_R_8_8	lPFtha, lateral pre-frontal thalamus	SubC	246	13, -16, 7
Tha, Thalamus / Tha_L_8_1	mPFtha, medial pre-frontal thalamus	SubC	232	-7, -12, 5
Tha, Thalamus / Tha_R_8_1	mPFtha, medial pre-frontal thalamus	SubC	231	7, -11, 6
PoG, Postcentral Gyrus / PoG_R_4_4	A1/2/3tru, area1/2/3(trunk region)	SMN	162	20, -33, 69
PCL, Paracentral Lobule / PCL_R_2_1	A1/2/3ll, area1/2/3 (lower limb region)	SMN	66	10, -34, 54

Note. Abbreviations, DAN = Dorsal Attention Network; VAN = Ventral Attention Network; SMN = Somatomotor Network; SubC = Subcortical regions.

Table S4. Correlation between confounding factors and predicted PSQI scores

Variables	Age	Gender	Education	Handedness	Race	Drug	Mean FD	BMI	TIV	Fluid Intelligence
r or T	0.0086	-1.2986	-0.0824	0.0334	-1.5603	1.5605	0.0463	-7.9×10^{-4}	-0.0672	0.0594
p	0.8589	0.1948	0.0861	0.4872	0.1194	0.1194	0.3356	0.9868	0.1621	0.2163

Table S5. Demographic characteristics of participants in HCP dataset (N=435)

Variables	No. (%)
PSQI, mean (SD)	4.68(2.73)
Age, mean (SD), years	28.65(3.69)
Female	230(52.87)
Handedness, mean (SD)[b]	66.63(42.39)
Education, mean (SD), years	14.91(1.76)
Race	
White	315(75.90)
Other	120(24.10)
Evidence of drug consumption on day of testing	67(15.40)
mean framewise displacement (FD), mean (SD)	0.15(0.05)
BMI, mean (SD), Kg/M^2	26.10(4.90)
Total intracranial volume (TIV), mean (SD), mm^3	1580344.55(188606.74)
Fluid Intelligence, mean (SD)[b]	16.98(4.80)

[a]Handedness of participant is assessed using the Edinburgh Handedness questionnaire.

[b]Fluid intelligence was measured by number of correct responses during the penn matrix test. This test measures fluid intelligence via non-verbal reasoning using an abbreviated version of the Raven's Progressive Matrices Form A developed by Gur and colleagues[26].

Table S6. Demographic characteristics of participants in the classification dataset

	Patients<br>Mean (SD)	HCs<br>Mean (SD)	T or X^2value	P value
**Dataset4**				
	N=52	N=49		
Age	44.00(12.39)	42.102(15.93)	0.6706	0.504
Sex	M:F=19:33	M:F=20:29	0.1948	0.6590
Mean FD	0.1437(0.0592)	0.1437(0.0615)	0.0001	0.9994
PSQI	12.96(3.81)[a]	4.49(2.74)[b]	11.67	1.44×10^{-19}
ISI	15.55(3.91)[c]	4.00(4.08)[d]	11.75	1.27×10^{-17}
**External Classification Dataset**				
	N=35	N=35		
Age	45.25(11.28)	40.83(9.27)	1.80	0.08
Sex	M:F=11:24	M:F=10:25	0.068	0.794
Mean FD	0.11(0.044)	0.11(0.046)	-0.074	0.941
PSQI	13.06(2.50)	3.66(2.45)	15.90	1.33×10^{-24}
ISI	16.49(4.51)	1.44(1.20)[e]	18.49	6.70×10^{-22}

[a]data available from 48 patients

[b]data available from 42 HCs

[c]data available from 33 patients

[d]data available from 33 HCs

[e]data available from 18 HCs

Note: Two sample t-test was conducted with an alpha level of 0.05 (two-tailed) to determine the difference of age mean FD, PSQI, ISI between insomnia patients and healthy controls (HCs). Chi-square test was performed with an alpha level

of 0.05 (two-tailed) to determine the difference of sex between patients and HCs. FD, Framewise displacement. PSQI, Pittsburgh Sleep Quality Index.

Table S7. Neurotransmitter receptors and transporters included in analyses

Receptor/ transporter	Neurotransmitter	Tracer	Measure	Age	Numbers	References
5-HT1a	serotonin	$[^{11}C]$WAY-100635	BP_{ND}	26.3 ± 5.2	35 (17)	[27]
5-HT1b	serotonin	$[^{11}C]$P943	BP_{ND}	28.7 ± 7.0	23 (8)	[27]
5-HT2a	serotonin	$[^{11}C]$Cimbi-36	B_{max}	22.6 ± 2.7	29 (14)	[28]
5-HTT	serotonin	$[^{11}C]$DASB	B_{max}	25.1 ± 5.8	100 (71)	[28]
$mGluR_5$	Glutamate	$[^{11}C]$ABP688	BP_{ND}	67.9 ± 9.6	22 (10)	PI: Rosa-Neto, P. & Kobayashi, E.
$GABA_{A/BZ}$	GABA	$[^{11}C]$Flumazenil	B_{max}	26.6 ± 8	16 (9)	[29]

Note: Values in parentheses (under n) indicate the number of females. BPND, non-displaceable binding potential; B_{max}, density (pmol ml−1) converted from binding potential (5-HT) or distributional volume (GABA) using autoradiography-derived densities.

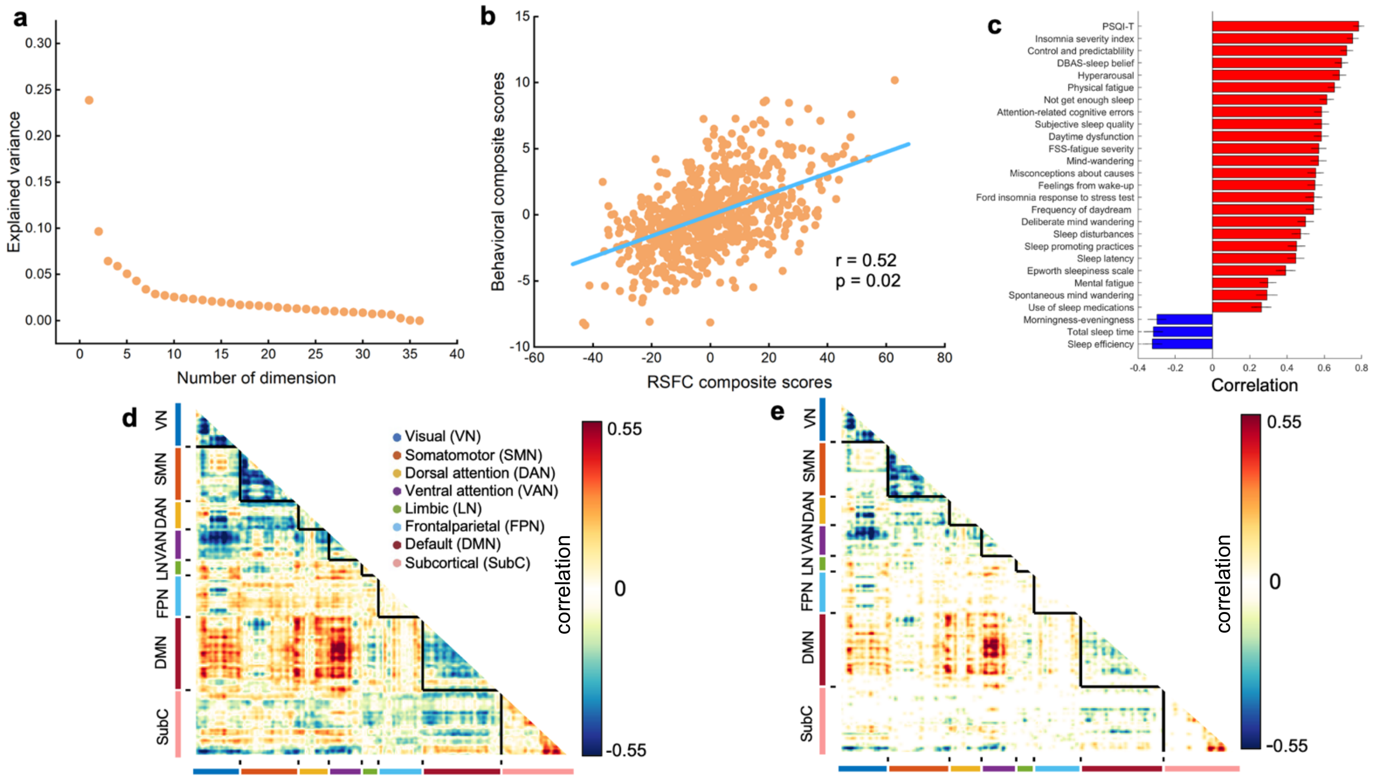

Fig S1. The PLS results remained largely unchanged when using a different Seitzman et al' Atlas [30] containing 300 regions for the RSFC construction. a, the amount of covariance explained by each latent variable (LV). Each orange dot represents a LV, only the first LV (LV1) survived after permutation testing with FDR correction (q<0.05). This survived dimension (LV1) accounted for 23.9% of resting-state functional connectivity (RSFC)-behavior covariance. b, scatter plots to illustrate the significant association between individual-specific resting-state functional connectivity (RSFC) and behavioral composite scores of participants in LV1(r =0.52, two-sided, permuted p = 0.02). c, top 27 strongest correlations between participants' behavioral measures and their behavioral composite scores on the group level. Greater loading on LV1 was associated with poorer sleep health. Error bars indicate bootstrapped standard deviation with 1000 bootstrap estimations (n=1000). Behavioral measures for which higher values indicate better sleep health are colored blue. For example, sleep efficiency is colored blue because higher values indicate better sleep quality. d, unthresholded correlations between participants' RSFC data and their RSFC composite scores. Red (or blue) color indicates that greater RSFC is positively (or negatively) associated with LV1. e, thresholded correlations between participants' RSFC data and their RSFC composite scores (false discovery rate q< .05). PSQI-T, total score of Pittsburgh Sleep Quality Index. DBAS, Dysfunctional beliefs and attitudes about sleep scale. Source data are provided as a Source Data file.

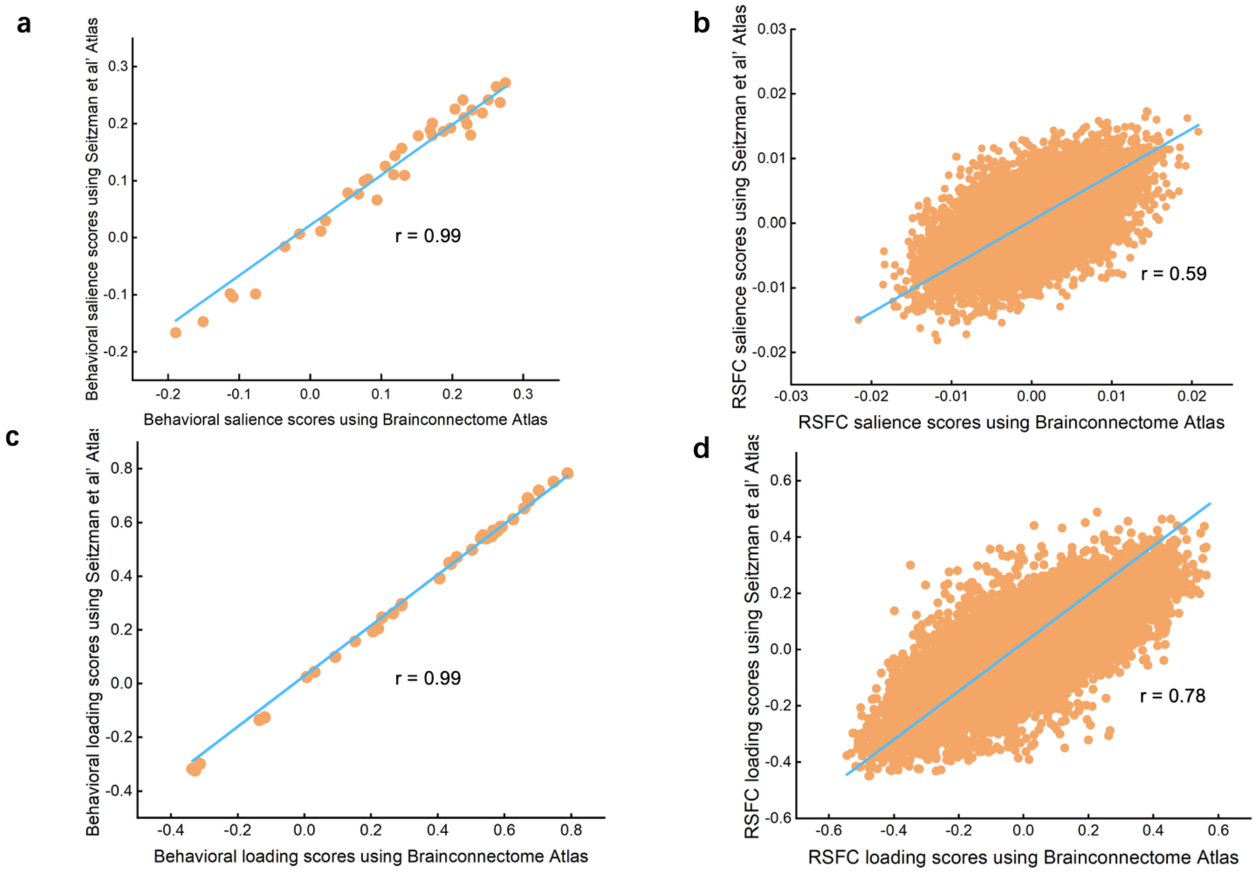

Fig S2. The robustness of the obtained LV1 was further ensured by using a different Seitzman et al' Atlas a, high correlation between salience scores of Brainconnectome Atlas and Seitzman et al' Atlas for the behavioral data using Pearson correlational analysis with an alpha level of 0.05 (two-tailed), r= 0.99, two-sided, p~=0. b, high correlation between salience scores of Brainconnectome Atlas and Seitzman et al' Atlas for the RSFC data using Pearson correlational analysis with an alpha level of 0.05 (two-tailed), r= 0.59, p~=0. c, high correlation between the loading scores of Brainconnectome Atlas and Seitzman et al' Atlas for the behavioral data using Pearson correlational analysis with an alpha level of 0.05 (two-tailed), r= 0.99, p~=0. d, high correlation between the loading scores of Brainconnectome Atlas and Seitzman et al' Atlas for the RSFC data using Pearson correlational analysis with an alpha level of 0.05 (two-tailed) , r~= 0.78, p~=0. LV, latent variable; RSFC, resting-state functional connectivity. Source data are provided as a Source Data file.

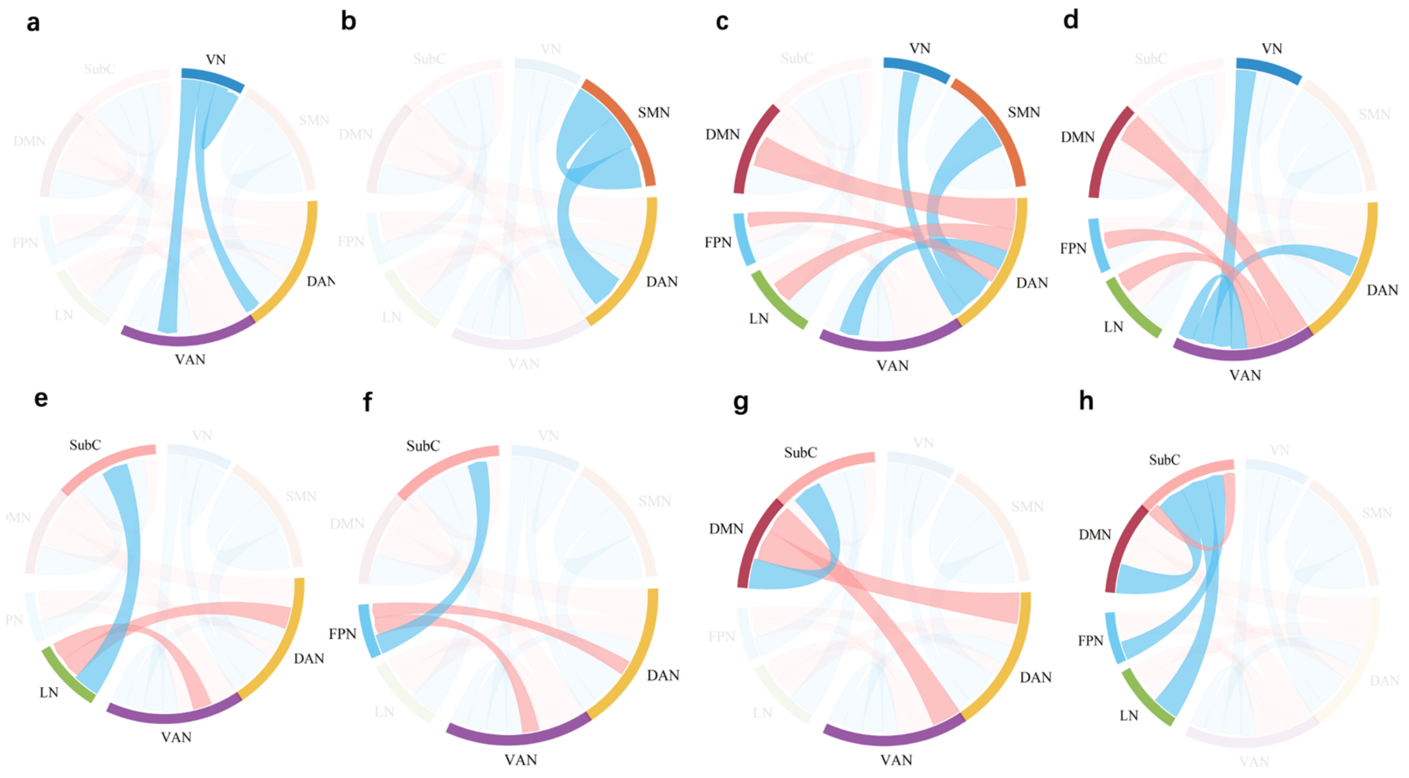

Fig S3. The significant RSFC correlations averaged within and between networks defined by Yeo et al's seven network. The pink line donates hyper-connectivity while the blue line donates hypo-connectivity. a, significant RSFC correlations with the visual network (VN). b, significant RSFC correlations with the somatomotor network (SMN).c, significant RSFC correlations with the dorsal attention network (DAN). d, significant RSFC correlations with the ventral attention network (VAN). e, significant RSFC correlations with the limbic network (LN). f, significant RSFC correlations with the frontoparietal network (FPN). g, significant RSFC correlations with the default mode network. h, significant RSFC correlations with the subcortical network (SubC). Source data are provided as a Source Data file.

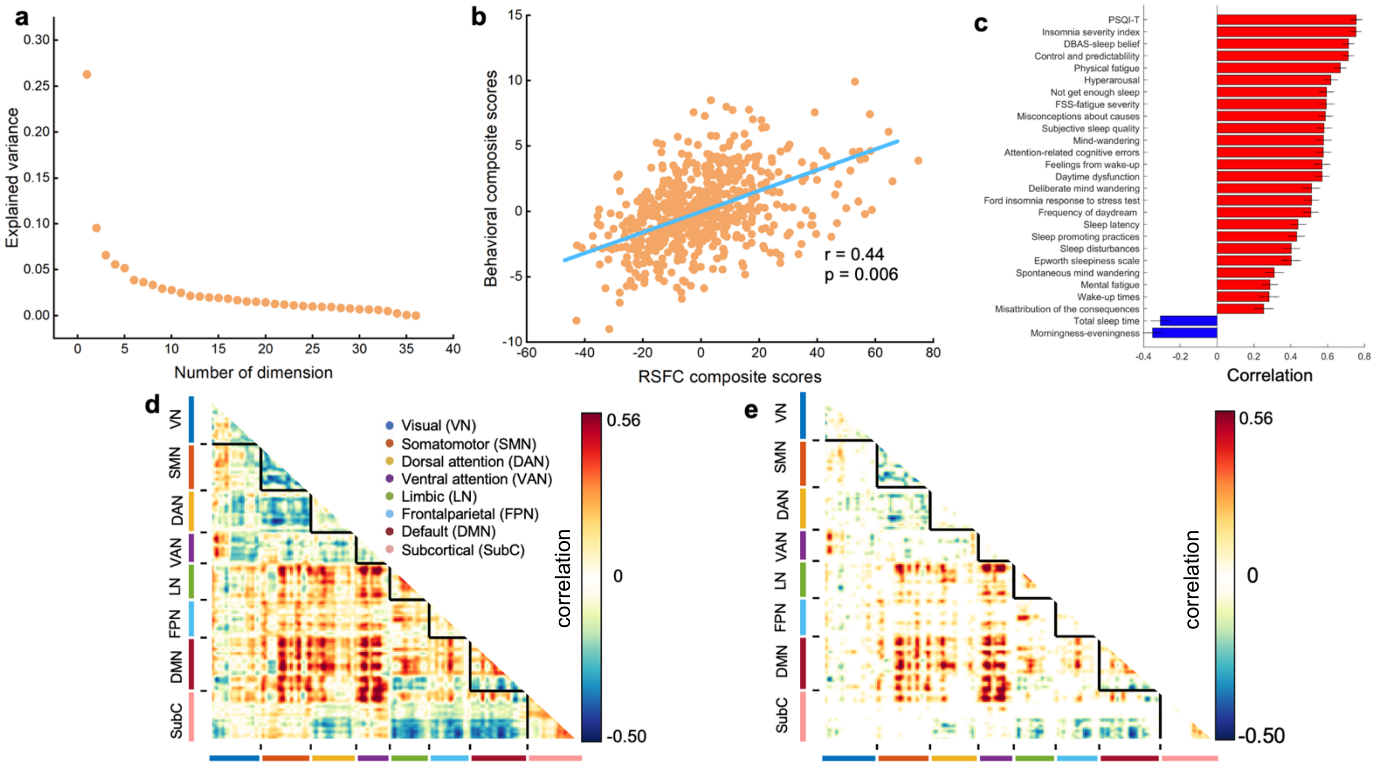

Fig S4. The PLS results was replicated with a large replication sample (N=628) from the BBP. a, the amount of covariance explained by each latent variable (LV). Each orange dot represents a LV, only the first LV (LV1) survived after permutation testing with FDR correction (q<0.05). This survived dimension (LV1) accounted for 26.3% of resting-state functional connectivity (RSFC)-behavior covariance. b, scatter plots to illustrate the significant association between individual-specific resting-state functional connectivity (RSFC) and behavioral composite scores of participants in LV1(r =0.44, two-sided, permuted p = 0.006). c, top 27 strongest correlations between participants' behavioral measures and their behavioral composite scores. Greater loading on LV1 was associated with poorer sleep health. Error bars indicate bootstrapped standard deviation with 1000 bootstrap estimations (n=1000). Behavioral measures for which higher values indicate better sleep health are colored blue. For example, sleep efficiency is colored blue because higher values indicate better sleep quality. d, unthresholded correlations between participants' RSFC data and their RSFC composite scores. Red (or blue) color indicates that greater RSFC is positively (or negatively) associated with LV1. e, thresholded correlations between participants' RSFC data and their RSFC composite scores (false discovery rate q< .05). PSQI-T, total score of Pittsburgh Sleep Quality Index. DBAS, Dysfunctional beliefs and attitudes about sleep scale. Source data are provided as a Source Data file.

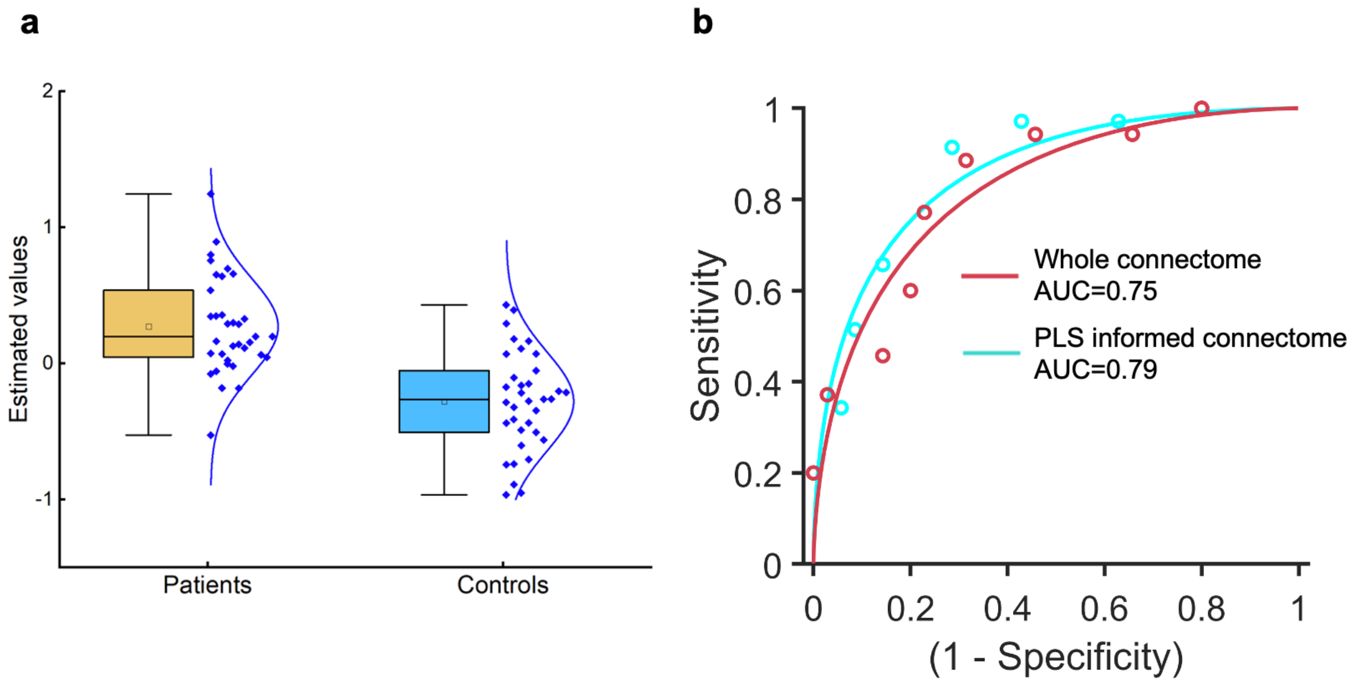

Fig S5. a. the mean estimated label values to index the classification performance for insomnia patients (n=35) and health controls (n=35) group across a 100 repeated 10-fold cross-validation strategy with the external classification dataset. b. the green receiver operating characteristic (ROC) curve depicts the classification performance using the significant edges of the RSFC spatial pattern obtained by PLS analysis in the external classification dataset, suggesting the sleep-health related connectome also has diagnostic potential to distinguish insomnia patients from sleep healthy subjects with an average accuracy across 100 CV of 78.29%, permuted $p = 3.89 \times 10^{-3}$; the red ROC curve depicts the classification performance using whole brain connectome with an average accuracy of 74.27%, permuted $p = 6.27 \times 10^{-3}$. LV, latent variable; RSFC, resting-state functional connectivity. Source data are provided as a Source Data file.

Supplementary References

1. Esteban, O. *et al.* fMRIPrep: a robust preprocessing pipeline for functional MRI. *Nat. Methods* **16**, 111–116 (2019).
2. Esteban, O. *et al.* fMRIPrep: a robust preprocessing pipeline for functional MRI (22.0.2). *Zenodo* (2022) doi:https://doi.org/10.5281/zenodo.7117719.
3. Gorgolewski, K. *et al.* Nipype: A flexible, lightweight and extensible neuroimaging data processing framework in Python. *Front. Neuroinform.* **5**, (2011).
4. Tustison, N. J. *et al.* N4ITK: Improved N3 bias correction. *IEEE Trans. Med. Imaging* **29**, 1310–1320 (2010).
5. Avants, B. B., Epstein, C. L., Grossman, M. & Gee, J. C. Symmetric diffeomorphic image registration with cross-correlation: Evaluating automated labeling of elderly and neurodegenerative brain. *Med. Image Anal.* **12**, 26–41 (2008).
6. Slagter, H. A. *et al.* PET Evidence for a Role for Striatal Dopamine in the Attentional Blink : Functional Implications. *J. Cogn. Neurosci.* **24**, 1932–1940 (2012).
7. Evans, A. C., Janke, A. L., Collins, D. L. & Baillet, S. Brain templates and atlases. *Neuroimage* **62**, 911–922 (2012).
8. Fonov, V. S., Evans, A. C., Mckinstry, R. C., Almli, C. R. & Collins, D. L. Unbiased nonlinear average age-appropriate brain templates from birth to adulthood. *Hum. Brain Mapp. J.* **47**, S102 (2009).
9. Jenkinson, M., Bannister, P., Brady, M. & Smith, S. Improved Optimization for the Robust and Accurate Linear Registration and Motion Correction of Brain Images. *Neuroimage* **17**, 825–841 (2002).
10. Cox, R. W. & Hyde, J. S. Software Tools for Analysis and Visualization of FMRI Data. *NMR Biomed. An Int. J. Devoted to Dev. Appl. Magn. Reson. Vivo* **10**, 171–178 (1997).
11. Jenkinson, M. & Smith, S. A global optimisation method for robust affine registration of brain images. *Med. Image Anal.* **5**, 143–156 (2001).
12. Greve, D. N. & Fischl, B. Accurate and robust brain image alignment using boundary-based registration. *Neuroimage* **48**, 63–72 (2009).
13. Power, J. D. *et al.* Methods to detect, characterize, and remove motion artifact in resting state fMRI. *Neuroimage* **84**, 320–341 (2014).
14. Behzadi, Y., Restom, K., Liau, J. & Liu, T. T. A component based noise correction method (CompCor) for BOLD and perfusion based fMRI. *Neuroimage* **37**, 90–101 (2007).
15. Satterthwaite, T. D. *et al.* An improved framework for confound regression and fi ltering for control of motion artifact in the preprocessing of resting-state functional connectivity data. *Neuroimage* **64**, 240–256 (2013).
16. Lanczos, C. Evaluation of Noisy Data. *J. Soc. Ind. Appl. Math. Ser. B Numer. Anal.* **1**, 76–85 (1964).
17. Abraham, A. *et al.* Machine learning for neuroimaging with scikit-learn. *Front. Neuroinform.* **8**, 1–10 (2014).
18. Wager, T. D., Lindquist, M. & Kaplan, L. Meta-analysis of functional neuroimaging data: Current and future directions. *Soc. Cogn. Affect. Neurosci.* **2**, 150–158 (2007).
19. Krishnan, A., Williams, L. J., McIntosh, A. R. & Abdi, H. Partial Least Squares (PLS) methods for neuroimaging: A tutorial and review. *Neuroimage* **56**, 455–475 (2011).
20. Courville, T. & Thompson, B. Use of structure coefficients in published multiple regression articles: β is not enough. *Educ. Psychol. Meas.* **61**, 229–248 (2001).
21. Sherry, A. & Henson, R. K. Conducting and interpreting canonical correlation analysis in personality research: A user-friendly primer. *J. Pers. Assess.* **84**, 37–48 (2005).
22. McIntosh, A. R. & Lobaugh, N. J. Partial least squares analysis of neuroimaging data: Applications and advances. *Neuroimage* **23**, 250–263 (2004).
23. Yarkoni, T. & Westfall, J. Choosing Prediction Over Explanation in Psychology: Lessons From Machine Learning. *Perspect. Psychol. Sci.* **12**, 1100–1122 (2017).
24. Malec, L. On the rank-deficient canonical correlation technique solved by analytic spectral decomposition. *J. Appl. Stat.* **49**, 819–830 (2022).
25. Woods, H., Marchetti, L. M., Biello, S. M. & Espie, C. A. The clock as a focus of selective attention in those with primary insomnia: An experimental study using a modified Posner paradigm. *Behav. Res. Ther.* **47**, 231–236 (2009).

26. Bilker, W. B. *et al.* Development of Abbreviated Nine-item Forms of the Raven's Standard Progressive Matrices Test. *Assessment* **19**, 354–369 (2015).
27. Savli, M. *et al.* Normative database of the serotonergic system in healthy subjects using multi-tracer PET. *Neuroimage* **63**, 447–459 (2012).
28. Beliveau, V. *et al.* A high-resolution in vivo atlas of the human brain's serotonin system. *J. Neurosci.* **37**, 120–128 (2017).
29. Nørgaard, M. *et al.* A high-resolution in vivo atlas of the human brain's benzodiazepine binding site of GABAA receptors. *Neuroimage* **232**, (2021).
30. Seitzman, B. A. *et al.* A set of functionally-defined brain regions with improved representation of the subcortex and cerebellum. *Neuroimage* **206**, 116290 (2020).
