# Supplementary material for: Covariance patterns between sleep health domains and distributed intrinsic functional connectivity
Source: Nat Commun. 2023 Nov 6;14:7133. doi: 10.1038/s41467-023-42945-5 (PMC10628193; doi:10.1038/s41467-023-42945-5)
Supplement: Supplementary file 3 — Reporting Summary [file 41467_2023_42945_MOESM3_ESM.pdf]

nature portfolio

Corresponding author(s): Xu Lei

Last updated by author(s): Sep 4, 2023

Reporting Summary

Nature Portfolio wishes to improve the reproducibility of the work that we publish. This form provides structure for consistency and transparency in reporting. For further information on Nature Portfolio policies, see our Editorial Policies and the Editorial Policy Checklist.

Statistics

For all statistical analyses, confirm that the following items are present in the figure legend, table legend, main text, or Methods section.

n/a	Confirmed	
☐	☒	The exact sample size (n) for each experimental group/condition, given as a discrete number and unit of measurement
☐	☒	A statement on whether measurements were taken from distinct samples or whether the same sample was measured repeatedly
☐	☒	The statistical test(s) used AND whether they are one- or two-sided<br>*Only common tests should be described solely by name; describe more complex techniques in the Methods section.*
☐	☒	A description of all covariates tested
☐	☒	A description of any assumptions or corrections, such as tests of normality and adjustment for multiple comparisons
☐	☒	A full description of the statistical parameters including central tendency (e.g. means) or other basic estimates (e.g. regression coefficient) AND variation (e.g. standard deviation) or associated estimates of uncertainty (e.g. confidence intervals)
☐	☒	For null hypothesis testing, the test statistic (e.g. F, t, r) with confidence intervals, effect sizes, degrees of freedom and P value noted<br>*Give P values as exact values whenever suitable.*
☒	☐	For Bayesian analysis, information on the choice of priors and Markov chain Monte Carlo settings
☒	☐	For hierarchical and complex designs, identification of the appropriate level for tests and full reporting of outcomes
☐	☒	Estimates of effect sizes (e.g. Cohen's d, Pearson's r), indicating how they were calculated

Our web collection on statistics for biologists contains articles on many of the points above.

Software and code

Policy information about availability of computer code

Data collection	Behavioral measures of sleep health and the demographical information were collected via Ding Ding (https://www.dingtalk.com/en) in the discovery and replication dataset. MRI data were acquired using validated standard protocols on a Siemens MRI system.
Data analysis	The preprocessing steps on all collected neuroimaging data in the discovery and replication dataset were performed using the publicly available CONN functional connectivity toolbox (version 20.b; https://www.nitrc.org/projects/conn), together with SPM12 (Wellcome Department of Cognitive Neurology, London, UK; http://www.fil.ion.ucl.ac.uk/spm). We used the Matlab code from https://github.com/danizoeller/myPLS, based on Krishnan and associates' work to implement the PLS calculation. The code for spatial permutation testing can be found at https://github.com/frantisekvasa/rotate_parcellation. The codes for SVR and SVM analysis are openly available at https://www.csie.ntu.edu.tw/~cjlin/libsvm/. The brain maps were presented using MRIcroGL toolbox (https://www.nitrc.org/projects/mricrogl). The connectome maps were presented using code from (https://github.com/cocoanlab/cocoanCORE/tree/master/Visualization).

For manuscripts utilizing custom algorithms or software that are central to the research but not yet described in published literature, software must be made available to editors and reviewers. We strongly encourage code deposition in a community repository (e.g. GitHub). See the Nature Portfolio guidelines for submitting code & software for further information.

Data

Policy information about availability of data

All manuscripts must include a data availability statement. This statement should provide the following information, where applicable:

- Accession codes, unique identifiers, or web links for publicly available datasets
- A description of any restrictions on data availability
- For clinical datasets or third party data, please ensure that the statement adheres to our policy

The HCP consortium database used in this study was freely available at the following accessible link: https://db.humanconnectome.org/. The BBP sample data are available under restricted access for it is still an ongoing project, access can be obtained by contacting a joint team (PIs: Q.H.H., J.Q., T.Y.F., H.C., and X.L.). The significant FC weights from the BBP discovery cohort are available at GitHub: https://github.com/wangyulinatUGent/Sleep_Health_Dimension. The classification sample used in this study were available upon request from the corresponding author. The PET/SPECT data from the prior vivo molecular imaging studies33 used in this study are available at the following accessible link: https://github.com/netneurolab/hansen_receptors/tree/main. Source data are provided with this paper.

Human research participants

Policy information about studies involving human research participants and Sex and Gender in Research.

Reporting on sex and gender	We included a sample of 687 participants (mean age 18.96, SD= 0.95; 233 males and 454 females) in the discovery sample, 628 participants (mean age 19.16, SD= 1.03; 208 males and 420 females) in the replication sample, 435 unrelated participants (mean age 28.65, SD= 3.69; 205 males and 230 females) in the HCP sample. The classification sample consisted of 52 ID patients (mean age= 44.00 years old, SD= 12.39, 19 males and 33 females) and 49 HC (mean age=42.10 years old, SD= 15.93, 20 males and 29 females). The external classification sample consisted of 35 ID patients (mean age= 45.25 years old, SD= 11.28, 11 males and 24 females) and 35 HC (mean age=40.83 years old, SD= 9.27, 10 males and 25 females). We did not specially consider sex in the design of the BBP project and collected both sexes. Sex was determined based on self-reporting. Before the PLS analysis, sex was regressed out as a confounder from both the RSFC and behavior data. Individual's sex information was provided in Table 1(we have also provided in the source data disaggregated sex data) and consent has been obtained to collect and share this information.
Population characteristics	The main dataset (the discovery and replication sample) comprises individuals from the Behavioral Brain Research Project of Chinese Personality (BBP), which was launched in September 2019 (still in progress) to recruit participants from that year's freshmen at Southwest University, Chongqing, China. For the discovery sample, the mean age is 18.96 years old, and it consists 233 males and 454 females. For the replication sample, the mean age is 19.16 years old, and it consists 208 males and 420 females. The HCP dataset provides high-quality behavioral/demographic and imaging data from healthy young adults (https://www.humanconnectome.org/)with a mean age of 28.65 years old, and it consists of 230 females and 205 males. The classification dataset involved 101 participants, including 52 patients(Mean age=44.0 years old, 33 females) with insomnia disorder (ID) and 49 healthy controls (HC, Mean age=42.10 years old, 29 females). The external classification dataset involved 70 participants, including 35 patients with ID (Mean age=45.25 years old, 25 females) and 35 HCs (Mean age=40.83 years old, 24 females).
Recruitment	The main dataset (the discovery and replication sample) comprises individuals from the Behavioral Brain Research Project of Chinese Personality (BBP), which was launched in September 2019 (still in progress) to recruit participants from that year's freshmen at Southwest University, Chongqing, China. The HCP consortium database was freely available at https://db.humanconnectome.org/. Part of the classification dataset was collected from the Sleep Center, Department of Brain Disease of Chongqing Traditional Chinese Medicine Hospital (CTCMH), which included 25 ID and 36 HC participants (referred as CTCMH dataset) and the remaining part of the classification dataset is from the Sleep and Neuroimaging Center, Southwest University (referred as SNIC dataset). The external classification dataset was collected at the Second Hospital of Hebei Medical University.
Ethics oversight	The current study complies with ethical regulations for research on human participants. The research projects were approved by the Southwest University(SWU) institutional review boards for the discovery and replication sample. Research procedures and ethical guidelines were followed in compliance with WU institutional review board approval. For the HCP sample, research procedures and ethical guidelines were followed in compliance with SWU institutional review board approval. For the classification sample, the research projects were approved by the SWU and CTCMH institutional review boards. For the external classification dataset, the research projects were approved by the review board of the Second Hospital of Hebei Medical University. Written informed consent was obtained from each participant in accordance with the Declaration of Helsinki.

Note that full information on the approval of the study protocol must also be provided in the manuscript.

Field-specific reporting

Please select the one below that is the best fit for your research. If you are not sure, read the appropriate sections before making your selection.

☒ Life sciences ☐ Behavioural & social sciences ☐ Ecological, evolutionary & environmental sciences

Life sciences study design

All studies must disclose on these points even when the disclosure is negative.

Sample size	The main discovery dataset (n=687) comprises individuals from the Behavioral Brain Research Project of Chinese Personality (BBP), the sample size was determined based on the inclusion and exclusion criterion related to our research question. Usually the sample size used for the PLS analysis is around 300, the sample size for the current study is sufficient. The robustness of the main findings was further replicated and generalized to several unseen independent datasets. Critically, the identified sleep-health related connectome showed diagnostic potential for insomnia disorder.
Data exclusions	For the discovery dataset, 25 participants were excluded due to excessive head motion during scanning (e.g., with a mean framewise displacement [FD] larger than 0.3 mm). For the replication dataset, 29 participants were excluded due to excessive head motion during scanning (e.g., with a mean framewise displacement [FD] larger than 0.3 mm). For the HCP dataset, 46 participants were excluded based on the exclusion criteria indicated as follows: (1) participants with missing values on demographic variables such as age, sex, education, BMI, and race or family information; (2) participants with a history of hyper/hypothyroidism or history of other endocrine problems; (3) women who had recently given birth; and (4) participants having a mean FD more than 0.3 mm. Importantly, to exclude the influence of shared genetic and environmental factors, we randomly kept one subject from each family, resulting in 435 final unrelated subjects. For the classification dataset, participants with any findings of pathological brain MRI as well as ineligibility for MRI scanning (any type of metal implant) were excluded in the study. Eight participants were excluded due to excessive head motion during the scanning (e.g., with a mean FD larger than 0.3 mm), resulting in a final sample of 52 ID patients and 49 HC.
Replication	We replicated the PLS procedure conducted in the discovery dataset((n=687)) with the replication dataset(n=628).
Randomization	Not applicable. The discovery and replication sample, as well as the HCP sample only included one group thus no randomization was performed. The classification dataset included a sample of 52 insomnia disorder patients and 49 health controls. Patients with insomnia disorder were diagnosed by experienced hospital psychiatrists according to the International Classification of Sleep Disorders: Diagnostic and Coding Manual, 3rd ed. and insomnia symptoms has lasted at least three nights a week for more than 3 months. To this end, no randomization was performed for the classification dataset.
Blinding	Given that the current study is resting-state and no between-subject design was employed, blinding is not relevant to the data collection and analysis.

Reporting for specific materials, systems and methods

We require information from authors about some types of materials, experimental systems and methods used in many studies. Here, indicate whether each material, system or method listed is relevant to your study. If you are not sure if a list item applies to your research, read the appropriate section before selecting a response.

Materials & experimental systems

n/a	Involved in the study
☒	☐ Antibodies
☒	☐ Eukaryotic cell lines
☒	☐ Palaeontology and archaeology
☒	☐ Animals and other organisms
☒	☐ Clinical data
☒	☐ Dual use research of concern

Methods

n/a	Involved in the study
☒	☐ ChIP-seq
☒	☐ Flow cytometry
☐	☒ MRI-based neuroimaging

Magnetic resonance imaging

Experimental design

Design type	Resting-state
Design specifications	During the resting-state scanning, a fixation cross was displayed as images were acquired. Participants were instructed to stay awake, keep their eyes open, fixate on the displayed crosshair, and remain still.
Behavioral performance measures	Given the present study is about resting-state fMRI, no behavioral performance measures were applied.

Acquisition

Imaging type(s)	functional and structural
Field strength	3 tesla (all datasets)
Sequence & imaging parameters	Functional MRI data for the discovery and replication sample was acquired using a blood oxygen level-dependent (BOLD-weighted) sequence (TR = 2000 ms; TE = 30 ms; slices = 62; slice thickness = 2 mm; FOV = 224 × 224 mm2; flip angle = 90°; resolution matrix = 112 × 112; voxel size=2× 2 ×2 mm3; phase encoding direction = PC » AC). Full details of the acquisition parameters for the HCP data can be found in the study of Kamil et al.,2013. Functional MRI data for the classification sample was acquired using an echo-planar imaging (EPI) sequence (TR/TE = 1500/29 ms, flip angle = 90°, resolution matrix = 64 × 64, voxel size = 3 ×3 ×3 mm3, FOV = 192 × 192 mm2, axial slices = 25, thickness/ gap = 5/0.5 mm).
Area of acquisition	A whole brain scan was used

Diffusion MRI ☐ Used ☒ Not used

Preprocessing

Preprocessing software	For the discovery and replication sample, the preprocessing steps were performed using the publicly available CONN functional connectivity toolbox (version 20.b; https://www.nitrc.org/projects/conn), together with SPM12 (Wellcome Department of Cognitive Neurology, London, UK; http://www.fil.ion.ucl.ac.uk/spm). For the HCP sample, we adopted the pre-processed data provided by the Human Connectome Project (HCP S1200 release). For the classification sample, the preprocessing steps on all collected neuroimaging data in the classification sample were performed using fMRIPrep 21.0.1.
Normalization	Nonlinear deformation based on anatomical data.
Normalization template	ICBM152 space.
Noise and artifact removal	Head motion parameters were removed.
Volume censoring	Outlier timepoints were included as nuisance regressors.

Statistical modeling & inference

Model type and settings	multivariate and predictive model
Effect(s) tested	multivariate relationships between resting state functional connectivity and sleep health;prediction of sleep health

Specify type of analysis: ☒ Whole brain ☐ ROI-based ☐ Both

Statistic type for inference (See Eklund et al. 2016)	non-parameter permutation test
Correction	FDR correction

Models & analysis

n/a	Involved in the study
☐	☒ Functional and/or effective connectivity
☐	☒ Graph analysis
☐	☒ Multivariate modeling or predictive analysis

Functional and/or effective connectivity	Pearson correlation
Graph analysis	Weighted graph
Multivariate modeling and predictive analysis	The current study applied PLS, an unsupervised machine learning technique that seeks to find covariance between two high-dimensional matrices, namely whole brain RSFC and 36 behavioral measures spanning multiple domains of sleep health; the current study then used support vector regression (SVR) to predict the sleep quality measured by the PSQI total score in the unrelated individuals of the HCP dataset; the current study also used a gaussian radial basis function (RBF) kernel support vector machine (SVM) classifier on our classification dataset. The significance of all the analysis were determined by the non-parameter permutation test.
